# Vulvar lactating adenoma associated to a fibroadenoma: common neoplasms in an uncommon site

**Published:** 2012-11-13

**Authors:** Amen Dhaoui, Haifa Nfoussi, Nidhameddine Kchir, Slim Haouet

**Affiliations:** 1 Pathology Department, La Rabta Hospital, Tunis El Manar University, Tunis, Tunisia

**Keywords:** Ectopic breast tissue, vulva, lactating adenoma, fibroadenoma, pathology

## Abstract

Ectopic breast tissue is defined as glands located outside of the breast. Ectopic breast tissue should be excised because it may develop benign (fibroadenoma) or malignant pathologic processes. Less than forty cases of fibroadenomas have been reported in the literature. Although lactation changes can occur, lactating adenoma in the vulva are extremely rare. Only four cases have been reported. We report a case of a young woman who presented with vulvar mass during her lactation. The mass was excised, and histology confirmed vulvar lactating adenoma associated with fibroadenoma. This is the first case of vulvar heterotopic breast lesion associating lactating adenoma and fibroadenoma.

## Introduction

Ectopic breast tissue is found along the primitive embryonic milk lines which extend from the axilla to the groin [1]. Incomplete involution anywhere along the primitive milk streak can result in ectopic mammary tissue. The ectopic vulvar breast tissue can give rise to neoplasia, both benign and malignant. But such cases are as a rarity [2]. Herein, we describe a case of accessory breast tissue presenting as a vulvar lactant adenoma associated to a fibroadenoma in a lactating woman [3].

## Patient and observation

A lactating 28-year old woman was referred to our institution complaining of an 3 month history of vulvar mass of progressive growth. Previous medical and familial history was not contributory to the present illness. A physical examination revealed a nontender, freely movable, 3 × 3 cm mass in labium major of the vulva. There was no sign of infection.

**Figure 1:**
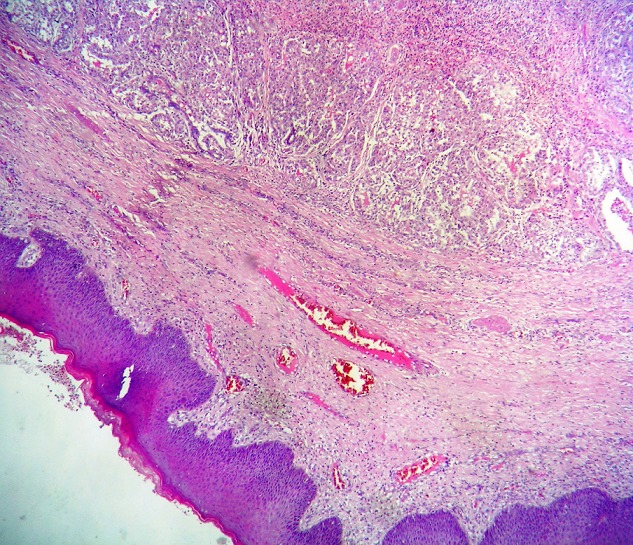
Dermal Lactating adenoma (H&E x40)

**Figure 2:**
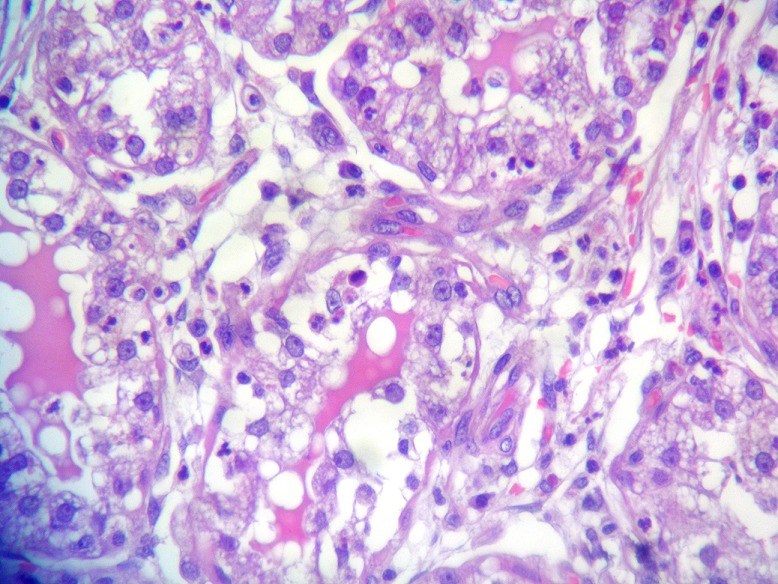
Secretory changes in lactating adenoma (H&E x400)

**Figure 3:**
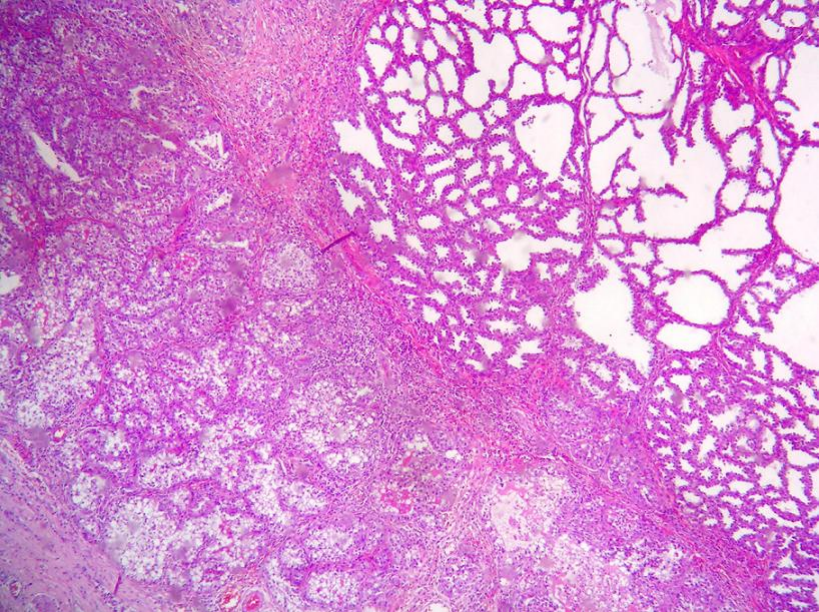
Glands are focally arranged in a lobular pattern similar to that in breast tissue and show cystic changes and apocrine-type secretion. (a H&E x40) (b H&E 200)

**Figure 4:**
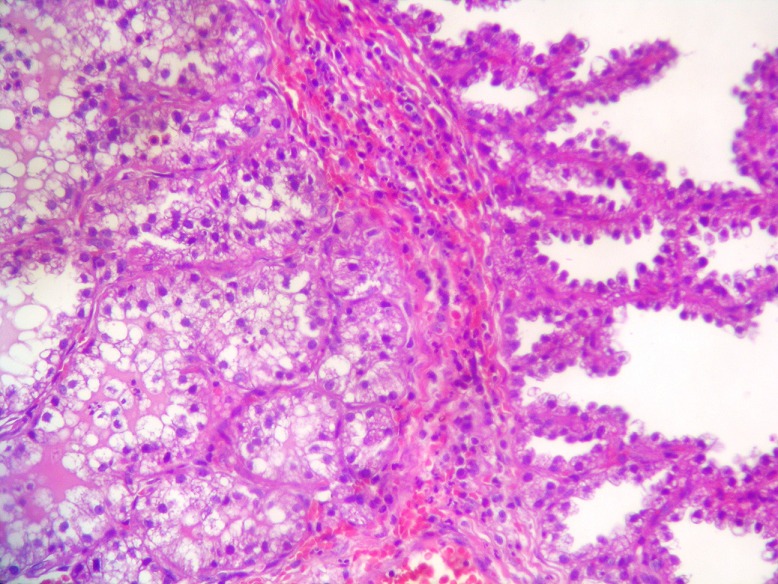
Vulvar glands organised on lobuli, resembling those of mammary glands

**Figure 5:**
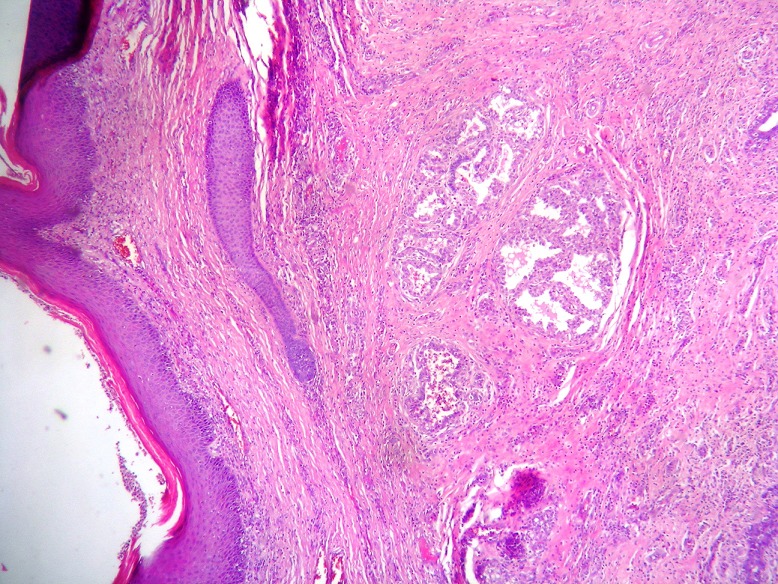
Dermal fibroadenoma(H&E x40)

**Figure 6:**
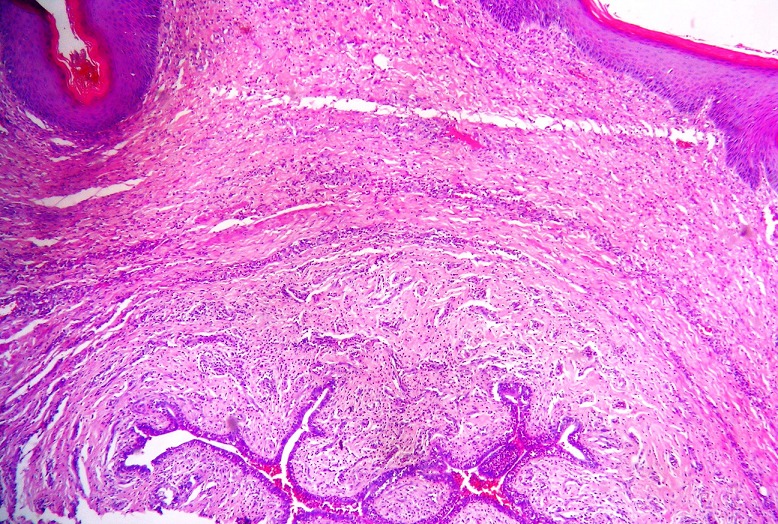
Depressed glands and ducts in dense fibrotic stroma resemble those in fibroadenoma of the breast (H&E x200)

A tumorectomy was performed. On gross examination, a well delimited multilobular mass with a skin ellipse was received. The measures of the mass were 3 x 3 x 2cm. Cut surface shows a lobulated white firm mass without necrosis or hemorrhage located in the dermis and subcutaneous tissue no related to the epidermis. Microscopic tissue sections revealed a typical lactating adenoma showing typical mammary glands with classic secretory activity, which included numerous intracytoplasmic lipid vacuoles, arranged in lobules with large alveolar spaces separated by fine fibrovascular trabeculae (Figure 1 and Figure 2). Some glands were dilated cystic or showing apocrine changes (Figure 3). Occasionally, glands were arranged in true lobuli, resembling those of mammary glands (Figure 4 and Figure 5). An architectural pattern similar to the typical pericanalicular mammary fibroadenoma was associated. It showed an admixture of stromal and epithelial component. The stromal cells proliferated around ducts in a circumferential fashion (Figure 6). The stromal pattern exhibited an extensive myxoid change. There was no atypia and mitoses were absent. The diagnosis of a vulvar lactant adenoma associated to a microscopic fibroadenoma rising in an ectopic mammary tissue with complete excision was made

## Discussion

Although the incidence of ectopic breast tissue is reported to occur in 2-6% of women, surprisingly there are only a few case reports in the literature, of breast tissue arising in the vulva [4]. This might be related to the fact that the vulva lies at the inferior end of the embryonic mammary ridge and therefore is a rare site for the location of ectopic mammary tissue. Ectopic breast tissue in the vulva was first reported in 1875 by Hartung. The ectopic breast tissue is subject to hormone responses and may develop benign and malignant pathologic processes similar to those seen in normally located breast tissue. About 50 cases of benign breast tissue in the vulva have been reported, typically in the 3rd and 4th decades of life, as well as over 20 cases of malignant lesions in women 40 to 82 years of age. Only half of malignant neoplasms were primary vulvar lesions. The others were metastatic from the breast. Authors have debated the origin of these lesions, including ectopic breast tissue, cutaneous apocrine glands and most recently, native anogenital mammary- like gland [2]. The presence of ectopic mammary tissue of normal characteristics surrounding the lesion described, such in our observation, supports the theory of ectopic mammary tissue.

Ectopic lactating adenoma has been noted as early as 23 and up to 39 years of age [3, 5]. Lactating adenoma are between 0, 8 and 4cm and the largest previously described was 7,8 cm [5]. Vular lactating adenoma is extremely rare; only four cases have been reported in the literature. The first manifestation reported of lactating vulvar adenoma, whether as tumefaction, was swelling or milk leakage through an injury [6]. Gynecomastia can be associated [6]. Vulvar fibroadenoma in uncommon, less than forty cases of vulvar fibroadenoma have been reported. Average patient age at moment of diagnosis of fibroadenoma was 38.7 years (range, 20-60 years), average tumor size was 3.0 cm (range, 0.8-6.0 cm) [9]. Fibroadenoma with adjacent ectopic breast tissue and fibroadenomas with lactational changes were reported in the literature but no case associating lactating adenoma was reported in vulva. To the best of our knowledge, our observation is the first case report presented in a lactating young woman with a microscopic fibroadenoma was associated to lactating adenoma in vulva and revealed on histological examination.

Pregnancy and lactatation superimpose profound morphologic changes on the so-called adenomas of the breast and ectopic breast-tubular and lactating adenomas and fibroadenomas [6–8]. Our patient was a lactating 28-years old woman. Histologically, ectopic lactating adenomas and adenofibroma are microscopically indistinguishable from those of the breast [9]. Vulvar lactating adenoma may be misdiagnosed as adenocarcinoma in frozen diagnosis and aspiration cytology if breast tissue is not anticipated [3]. Ectopic breast tissue is able to undergo malignant transformation; therefore, complete excision of symptomatic breast tissue found in the vulva should be considered [1].

## Conclusion

Vulvar heterotopic breast is rare and can present a challenge for both the clinician and the anatomical-pathologist in making the correct diagnosis. Diagnosis is confirmed by biopsy. Neoplasms rising in this accessory mammary tissue are extremely rare. To the best of our knowledge, our observation is the first case report with a lactating adenoma associated to microscopic fibroadenoma and revealed on histological examination.
